# Supplementary figures and images for: Production and Characterization of Rhamnolipids Produced by Pseudomonas aeruginosa DBM 3774: Response Surface Methodology Approach
Source: Microorganisms. 2022 Jun 22;10(7):1272. doi: 10.3390/microorganisms10071272 (PMC9321515; doi:10.3390/microorganisms10071272)

## Supplementary Materials

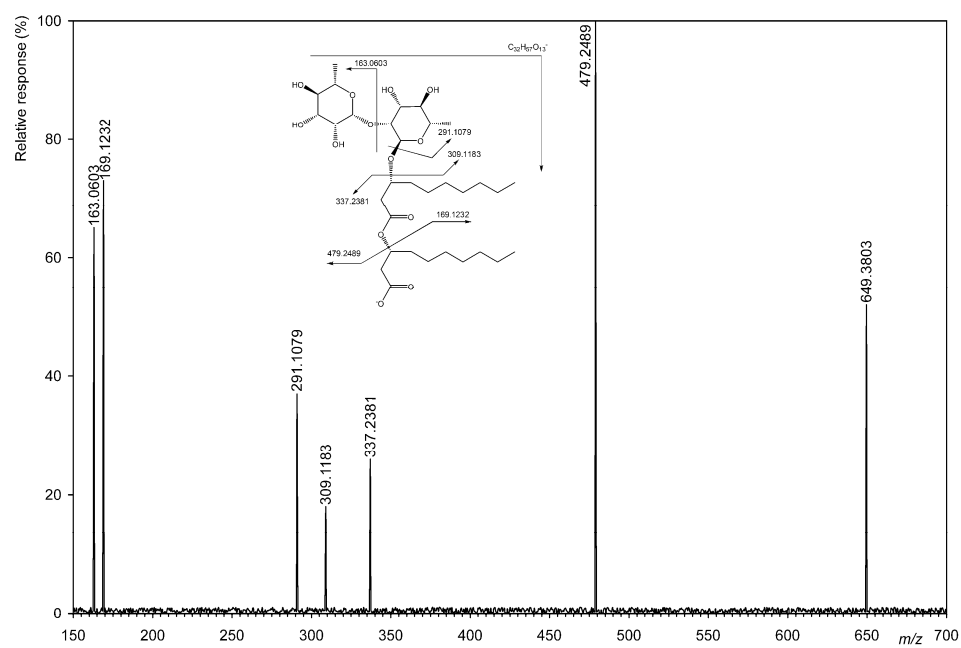

**Figure S1.** Tandem mass spectrum of most abundant rhamnolipid (RhaRha1010).

Supplement: Supplementary file 1 [file microorganisms-10-01272-s001.zip › microorganisms-1771653-supplementary.pdf]
